# Outcomes of the 2019 hydrocephalus association workshop, "Driving common pathways: extending insights from posthemorrhagic hydrocephalus"

**DOI:** 10.1186/s12987-023-00406-7

**Published:** 2023-01-13

**Authors:** Jason K. Karimy, Jessie C. Newville, Cameron Sadegh, Jill A. Morris, Edwin S. Monuki, David D. Limbrick, James P. McAllister II, Jenna E. Koschnitzky, Maria K. Lehtinen, Lauren L. Jantzie

**Affiliations:** 1 Department of Family Medicine, Mountain Area Health Education Center - Boone, North Carolina, 28607 USA; 2 Department of Pediatrics and Neurosurgery, Johns Hopkins Children’s Center, Johns Hopkins School of Medicine, Baltimore, MD 21287 USA; 3 Department of Neurosurgery, Massachusetts General Hospital and Harvard Medical School, MA Boston, 02114 USA; 4 National Institute of Neurological Disorders and Stroke, Neuroscience Center, National Institutes of Health, 6001 Executive Blvd, NSC Rm 2112, Bethesda, MD 20892 USA; 5 Departments of Pathology & Laboratory Medicine and Developmental & Cell Biology, University of California Irvine, Irvine, CA 92697 USA; 6 Departments of Neurosurgery and Pediatrics, Washington University School of Medicine in St. Louis, St. Louis, MO 63110 USA; 7 Hydrocephalus Association, Bethesda, MD 20814 USA; 8 Department of Pathology, Boston Children’s Hospital, Boston, MA 02115 USA; 9 Kennedy Krieger Institute, Baltimore, MD 21287 USA

**Keywords:** Hydrocephalus, Posthemorrhagic, Post-infectious, Intraventricular hemorrhage, Germinal matrix hemorrhage, Cilia, Ependyma, Cerebrospinal fluid

## Abstract

The Hydrocephalus Association (HA) workshop, Driving Common Pathways: Extending Insights from Posthemorrhagic Hydrocephalus, was held on November 4 and 5, 2019 at Washington University in St. Louis. The workshop brought together a diverse group of basic, translational, and clinical scientists conducting research on multiple hydrocephalus etiologies with select outside researchers. The main goals of the workshop were to explore areas of potential overlap between hydrocephalus etiologies and identify drug targets that could positively impact various forms of hydrocephalus. This report details the major themes of the workshop and the research presented on three cell types that are targets for new hydrocephalus interventions: choroid plexus epithelial cells, ventricular ependymal cells, and immune cells (macrophages and microglia).

## Background

The Hydrocephalus Association hosted a workshop at the Charles F. Knight Center on November 4 and 5, 2019 at Washington University in St. Louis. The mission of the Hydrocephalus Association is to find cures for hydrocephalus and improve the lives of those affected by the condition. The Association strives to accomplish this mission by collaborating with patients, caregivers, researchers, and industry, raising awareness, and funding innovative, high impact research to prevent, treat, and ultimately cure hydrocephalus. In 2009, the Hydrocephalus Association developed a research program and is now the largest non-governmental funder of hydrocephalus research in the United States, committing over $11 million to research during the past 10 years.

Hydrocephalus is a condition characterized by a dysfunction in the balance of production and absorption of cerebrospinal fluid (CSF) within the brain and cranial cavity. The incidence of hydrocephalus is difficult to calculate, especially in the adult population [1, 2]. However, a recent study estimated the worldwide incidence of pediatric hydrocephalus at nearly 400,000 cases per year, with 5,000 of those cases occurring in the United States [1]. The primary treatment for hydrocephalus is placement of a permanent shunt that drains CSF from the ventricles to another location in the body. For certain indications, endoscopic third ventriculostomy (ETV) or ETV with choroid plexus cauterization (ETV-CPC) may be used [3]. Despite efforts to reduce treatment failure, the rates remain unacceptably high over a patient’s lifetime and no non-surgical treatments are available.

This workshop, *Driving Common Pathways: Extending Insights from Posthemorrhagic Hydrocephalus (PHH)*, was developed through the Hydrocephalus Association’s PHH Initiative, a multiyear endeavor focused on increasing research into the condition. The workshop agenda was developed by a planning committee including basic, translational, and clinical researchers and was led by Hydrocephalus Association staff. The workshop expanded on discussions held at the 2016 Hydrocephalus Association PHH Workshop, which was held at the National Institutes of Health [4]. Recent publications have shown significant overlap between mechanisms implicated in PHH and other etiologies of hydrocephalus [5–11]. However, despite the apparent overlaps, much of this research has been siloed.

The pathophysiology of hydrocephalus can be incredibly multifactorial, with common genes, molecular pathways, and cellular alterations. The goal of this workshop was therefore to provide a small group setting in which researchers could (1) challenge preconceptions, (2) explore commonalities and differences between hydrocephalus etiologies, (3) identify potential treatments and therapies applicable to multiple etiologies, and (4) build collaborations. By bringing researchers together who study similar mechanisms in different models and species, collaborations can be developed to validate drug targets across etiologies and increase the potential impact of this research.

The workshop began with overviews on clinical research and overlapping disease mechanisms. Day One sessions were focused on the choroid plexus, cilia, brain development, and microglia. Day Two began with an overview of neuroinflammation and drug targets followed by a session focused on ependyma and ending with an exploration of opportunities in cell therapies. The workshop closed with a discussion focused on opportunities and next steps. The full agenda can be found here: https://www.hydroassoc.org/sponsored-research-events/.

**Fig. 1:**
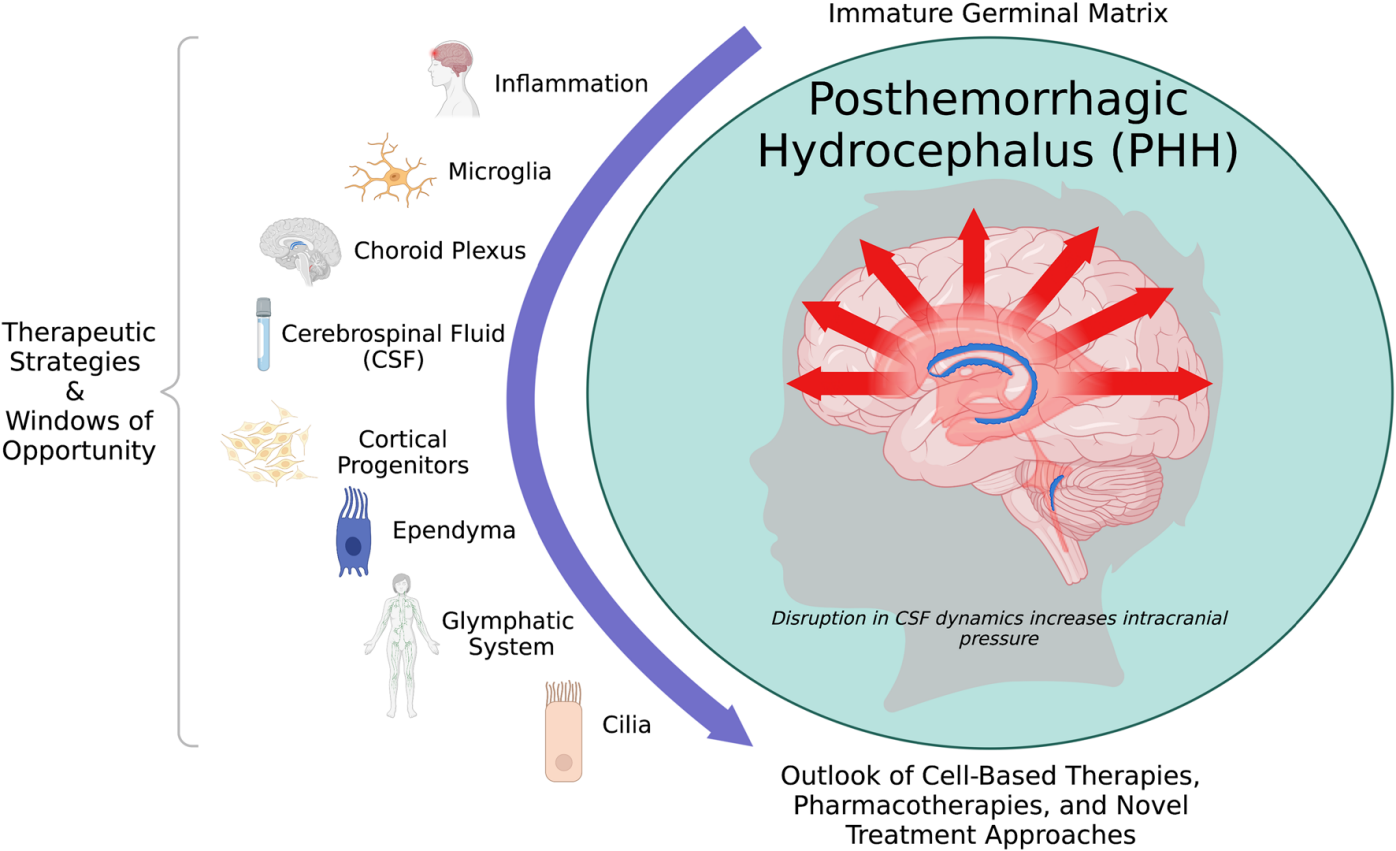
Posthemorrhagic hydrocephalus of prematurity is a complex medical condition with intricate, developmentally regulated pathophysiology. Defined by disruption to cerebral spinal fluid dynamics through unique cellular and molecular mechanisms, novel, non-surgical treatment approaches address inflammation, fluid dynamics, choroid plexus, ependyma, cilia, and the glymphatic system in concert. Created by Biorender.com

This report reviews the presented research and major themes discussed during the *Driving Common Pathways: Extending Insights from Posthemorrhagic Hydrocephalus* workshop (Figure 1). The views and recommendations in this report were developed by the listed authors based on workshop discussions and integrated into the report.

## Hydrocephalus etiologies

The workshop began with a clinical overview of hydrocephalus starting with a review of PHH of prematurity. PHH of prematurity accounts for 20% of shunted pediatric hydrocephalus cases in the United States [12] and is a particularly devastating etiology with significant morbidity and mortality [13]. Children with PHH of prematurity have high shunt complication rates and longer hospital stays than children with other forms of the condition [13, 14]. Most of these children will also develop a major disability such as cerebral palsy, epilepsy, intellectual disability, and neurobehavioral impairments [15–18]. As a result, PHH of prematurity has a negative impact on family function [14, 19] as well as the affected individual’s quality of life [20].

Risk factors for and the pathophysiology of PHH of prematurity have been reviewed elsewhere [4, 21−23]. Briefly, PHH of prematurity is caused by the rupture of blood vessels within the germinal matrix, a transient anatomical region in the developing human brain home to neural and glial precursor cells. Extravasation of blood into the ventricles constitutes intraventricular hemorrhage (IVH) and can lead to PHH as well as other lifelong neurological sequelae [21, 24−26]. Premature infants are particularly susceptible to IVH. Indeed, the risk of IVH is inversely related to gestational age and corresponds with the development and subsequent decline of the germinal matrix. By gestational week 37, the germinal matrix is nearly absent during normal development [21, 27−30]. The risk of developing PHH of prematurity following IVH is related to the severity and extent of the hemorrhage as defined by the Papile scale (Grade I-IV), with Grade IV being the most severe [31] 75% of the neonates who develop PHH have a Grade III or Grade IV IVH [13].

However, PHH of prematurity is one of many hydrocephalus etiologies, and this workshop sought to broaden the impact of research funded through the PHH Initiative and break down research silos. Hydrocephalus is typically defined by the causative factor, age of onset, and/or type of CSF dysfunction, and etiologies are broadly grouped into one of four major categories: congenital hydrocephalus, acquired hydrocephalus, idiopathic Normal Pressure Hydrocephalus (iNPH), or idiopathic intracranial hypertension. However, these categories are not exclusive. Depending on context and gestational age, PHH of prematurity has been included in both the acquired and congenital categories for instance. In addition, although PHH of prematurity often occurs in a setting where infection is present, it is nonetheless distinct from post-infectious hydrocephalus (PIH) [32–34].

Congenital hydrocephalus is generally diagnosed at or near birth and is often, but not always, due to brain structural abnormalities that block the flow of CSF within the ventricular system. Congenital hydrocephalus can be part of a known syndrome or condition with identified genetic and environmental factors, although many cases are idiopathic in nature. Recent work [35] has begun to identify genetic mutations linked to the development of cases formerly classified as idiopathic.

Acquired hydrocephalus can develop any time after birth and results from a known cause that increases CSF production, blocks the flow of CSF within the ventricular system or subarachnoid spaces, and/or alters the absorption of CSF. PHH and PHH of prematurity fall into the acquired category due to traumatic brain injury, stroke, and other causes of brain hemorrhage. PIH and tumor-associated hydrocephalus also fall into this category. However, hemorrhage, infection, and tumor that occur *in utero* and result in the development of hydrocephalus are often classified as congenital hydrocephalus.

Idiopathic Normal Pressure Hydrocephalus (iNPH) is a condition typically associated with adults over 60 years of age and is characterized by the presence of enlarged ventricles and intracranial pressures that fluctuate between normal and abnormal values. The pathogenesis of iNPH is largely unknown. Most theories are focused on disturbances in CSF dynamics that interplay with vascular, metabolic, neurodegenerative, and hereditary factors [36, 37]. iNPH can also occur due to a known cause such as a brain hemorrhage or infection, although these cases are classified as an acquired form of the condition.

As detailed above, hydrocephalus can develop at any age from a wide variety of causes. This complexity can complicate the development of new therapies to prevent, treat, or modify the condition but also affords us the possibility of discovering common genes, molecular pathways, and cellular alterations critical to the development of hydrocephalus across etiologies. Throughout the workshop, participants discussed both unique and converging mechanisms (i.e., inflammation) as well as the role age of onset plays in selecting appropriate therapeutic targets. Timelines for interventions can also influence hydrocephalus’ impact on the brain and alter the many co-morbidities that can complicate clinical care.

## Core mechanisms

Fundamentally, hydrocephalus involves an altered fluid balance that entails excess accumulation and changes in CSF dynamics in the brain. In turn, CSF accumulation triggers an array of downstream injuries (i.e., white matter injury, changes in neural stem cell populations) that contribute to the constellation of neurological sequelae associated with hydrocephalus [24, 38, 39].Therefore, the removal of CSF either via surgical shunts or by endoscopic third ventriculostomy has proven remarkably successful in terms of addressing a primary feature of hydrocephalus, relieving intracranial pressure, and limiting the extent of secondary injuries [40–44]. However, as surgical interventions are invasive and fraught with complications, new therapeutic advances, including non-surgical approaches, are desperately needed [43]. To begin making steps towards improving treatment options for all forms of hydrocephalus, more information is needed regarding even the very basic mechanisms of CSF production and removal at baseline. Hence, a key question in the field - *What causes excess CSF accumulation in the brain -* has multiple answers, reflecting the multi-factorial nature of hydrocephalus.

The main source of CSF is the choroid plexus, an epithelial sheet located in each ventricle in the brain, though extra-choroidal sources of CSF including cerebral microvasculature certainly exist. At the choroid plexus, CSF-water is sourced from the systemic circulation. Water channels (Aquaporins), ion co-transporters (e.g., NKCC1, see below), and other channels (e.g., TRPV4) are considered to be important contributors to CSF’s water content [11, 45−47]. Once secreted, CSF fills the ventricular system and central canal of the spinal cord; it also enters the subarachnoid space. CSF is resorbed into the systemic circulation at various possible exit points along this path including nerve roots around the cribriform plate, lymphatic vessels, and potentially arachnoid granulations (not present in lower mammalian species) [48]. During this process, CSF can exchange with interstitial fluid and clear metabolites and waste from the parenchyma (also called the glymphatic system) [49]. While the choroid plexus is traditionally viewed as the main source of CSF, recent evidence also points to the possibility that under certain conditions, bi-directional transporters at the choroid plexus can also remove CSF-water from the ventricles [50]. Thus, as disease mechanisms underlying hydrocephalus traditionally involve excess secretion, obstruction of flow, or impaired CSF exit from the brain, a multitude of possibilities emerge as potential target sites that trigger the condition. Regardless of disease etiology, once CSF accumulates, expansion of the ventricles imparts substantial mechanical stress on the brain.

The health and function of the choroid plexus, ependyma and motile cilia, and glymphatic system are interwoven. There is a well-documented association between prenatal blood-borne inflammation and posthemorrhagic hydrocephalus of prematurity. Significantly, people of all ages with IVH that progresses from ventriculomegaly to hydrocephalus are more likely to have sepsis [51, 52]. Notably, neuroinflammation may reduce propulsion of CSF by motile cilia [53]. Similarly, sustained inflammation impacts the homeostatic composition of trophic and toxic factors secreted and released into the CSF. The associated downstream signal transduction, including the production of cytokines and chemokines, is central to inflammatory regulation, immune cell recruitment, glial activation, and neural-immune injury [5, 54].

## Choroid plexus epithelium

The first session of the workshop began with a thorough discussion of the choroid plexus epithelium (CPe), its form and function, and described novel mechanisms of ion flux and interplay with the immune system that support its role in multiple etiologies of hydrocephalus [55–58].Historically, the choroid plexus was primarily implicated in hydrocephalus in the context of choroid plexus papilloma and villous hypertrophy via CSF overproduction [59] or in clinical studies involving ETV-CPC [60, 61]. However, recent studies have begun demonstrating overlooked functions of the choroid plexus that may provide therapeutic targets in PHH. The choroid plexus session put forth innovative data and fostered discussions focused on novel roles of the choroid plexus in the development of hydrocephalus. During this session, topics introduced included the regulation and dysregulation of ion flux, the response of the choroid plexus to blood-breakdown products, evidence of CSF hypersecretion in the context of posthemorrhagic, post-infectious, and congenital hydrocephalus, and the emerging role of the choroid plexus in CSF reabsorption. During these discussions, it was abundantly clear that pathophysiology of choroid plexus has been historically understudied and further characterization will likely provide a fruitful avenue for the development of non-invasive therapeutics across multiple etiologies of hydrocephalus.

### Development and function of the choroid plexus

The CPe comprises a highly vascularized network of fenestrated capillaries surrounded by polarized cuboidal secretory epithelial cells linked via tight junctions [45, 62−64]. The CPe is an anchored structure within each cerebral ventricle extending in continuity from the ependymal lining of the ventricular walls. The CPe emerges at approximately the seventh week of gestation when it begins to perform essential functions in neurodevelopment. Classically, the CPe is viewed as the primary site of CSF secretion [58], which is tightly regulated by a network of ion-transporters, kinases, and the osmotically driven movement of water through aquaporins. Maintenance of CSF volume homeostasis is critical to normal brain development as inadequate intracranial pressure can impair brain folding and development [65–67]. Abnormal accumulation of CSF increases intracranial pressure, compressing surrounding periventricular structures and impairing brain function [59]. CPe functions beyond CSF production are often overlooked. The CPe is also the site of the blood-CSF barrier, and it performs critical roles in neurohormonal modulation and immune surveillance, particularly during times of injury [54, 55]. CPe sites also have unique molecular profiles within each ventricle [68, 69], suggesting subspecialized functions for different brain regions [70, 71]. As studies in the field continue, we will likely find more roles of this complex and critical structure.

### Unique epithelial structure

In general, epithelial cells exhibit polarity via asymmetric distribution of membrane proteins between the apical and basolateral membranes [58]. Polarity allows epithelial cells to establish cellular barriers and vectorial transport critical to multiple regulatory processes. Unlike most secretory epithelial cells outside of the brain, the CPe has many ion co-transporters, such as Na^+^/K^+^ ATPase, the Na^+^/H^+^ exchanger 1 (NHE1) and NKCC1, expressed on the apical membrane instead of the basolateral membrane. This reverse polarity is incompletely understood but may provide insight into CPe function and its response to injury and treatment. For example, as amiloride, NHE and ENaC (Epithelial Na^+^ channel) transport blocker, has only been demonstrated to effectively modulate CSF secretion if given on the basolateral (blood-side) of the CPe, its mechanism of action is thought to selectively involve basolateral sodium transport [45, 72]. Whereas, other drugs such as ouabain, furosemide, and bumetanide only modulate CSF secretion when administered on the apical (ventricular) side. This may explain the failure of furosemide in clinical trials for PHH [73, 74]. Given the importance of bicarbonate co-transport, cytosolic carbonic anhydrase can be inhibited by acetazolamide from both apical and basolateral sides to decrease CSF secretion. However, in the context of PHH, acetazolamide was not effective in attenuating CSF hypersecretion [32]. Findings like these indicate that the CPe is tightly regulated and while some therapeutic targets may have very strong findings in experimental models, they pose challenges for translational patient care.

### Dysregulation of ion flux across the CPe

In this workshop, special attention was given to discussing ion and water transport across the CPe, given the perturbations in fluid homeostasis following PHH and other etiologies of hydrocephalus. The ion-cotransporter NKCC1 and the transient receptor potential vanilloid 4 (TRPV4) attracted particular interest due to recent advances made by several groups [8, 10, 11, 46, 50, 59, 75−77]. NKCC1 is an electroneutral Na^+^/ K^+^/2Cl^−^ ion co-transporter that is highly expressed on the apical side of CPe epithelial cells. Of relevance to CSF secretion, NKCC1 transport direction depends on the combined electrochemical gradients for Na^+^, K^+^ and Cl^−^, and water follows the movement of these ions. Although NKCC1 is a bidirectional transporter, its transport direction and details underlying water movement across the choroid plexus have generated a lively discussion for the field [78]. Early studies suggested that in a majority of circumstances, NKCC1 transports ions inward (into the CPe, reviewed by Gregoriades et al. 2019) [75, 79]. Recent in vivo findings in developing mouse brain showed that choroid plexus-NKCC1 overexpression is accompanied by reduced ventricle size [50]. However, it is important to note that these studies were performed around birth in mouse, a developmental stage when CSF-K^+^ was found to be substantially higher (9.6 mM ± 3.5) than adult CSF-K^+^ levels (3.1 mM ± 0.6) [50]. In adult brain, work by Steffensen et al. [80]. suggests that the directionality of transport at the choroid plexus can be outward (towards the ventricles) for ions and water. In this latter model, CSF secretion occurs actively via co-transport of ions and water, and independently of conventional osmosis [81]. Thus, recent research on NKCC1 has uncovered fundamental questions for the field regarding our understanding of transport of ions and water, both which are technically challenging to track in real-time in vivo [50, 58, 75, 78, 79, 82−86]. As such, the mechanisms by which NKCC1 and other ion co-transporters participate in water movement and hydrocephalus represent a vibrant area of research.

Calcium ion regulation has also been implicated as a contributor to CSF secretion and is postulated to have a role in hemorrhagic injury and the accompanying dysregulation of ion-flux. These mechanisms have been recently shown to be largely mediated by TRPV4, which is a calcium-permeable non-selective ion transporter abundantly found on the apical membrane of the CPe [76, 87, 88]. TRPV4 activation in human choroid plexus epithelial cells stimulates fluid secretion in vitro [89]. TRPV4 has also been implicated in ischemic stroke, edema, and astrocytic swelling [90] as a driver of fluid movement and represents an interesting therapeutic target given the critical role of calcium as a secondary messenger in many signaling pathways. This transporter is activated by mechanical stress [91], which is suspected in the setting of high ICP, ventriculomegaly, and hydrocephalus. Moreover, TRPV4 is regulated by inflammatory cytokines which have consistently been observed in PHH and PIH [5, 10]. Therefore, targeting this transporter may complement and/or synergize with other therapeutic approaches that target the modulation of NKCC1 [11, 50, 77, 86]. The potential role for TRPV4 and inflammation is discussed below.

### Inflammation and potential intervention sites

The role of inflammation in PHH and other etiologies of hydrocephalus has been postulated and studied for some time [5]. During this workshop, investigators discussed inflammatory responses to hemorrhage and the consequent blood-breakdown products. Parenchymal microglia, resident macrophages within the CPe, other barrier-associated macrophages (in the meninges and perivascular spaces), and choroid plexus epithelial cells contribute to active immune surveillance of the ventricular system. These cells bind damage-associated molecular patterns (DAMPs) and pathogen-associated molecular patterns (PAMPs) via toll-like receptors which in multiple models leads to an increase in pro-inflammatory cytokines associated with PHH [5]. For example, in an in vivo rodent model of IVH, the activation of CD68^+^ macrophages is dependent on the TLR4-NF-κB (Toll-like receptor 4 – nuclear factor kappa-light-change-enhancer of activated B cells) pathway [32]. NF-κB is a transcription factor downstream of TLR4 and has been implicated in the release of pro-inflammatory cytokines TNF-α (tumor necrosis factor-alpha) and IL-1β (interleukin-1β). At present, it appears that these cytokines alter the function of the multiple ion-transporters on the choroid plexus. For example, blood stimulates a TLR4-NF-κB-mediated response including a change in the kinetics of NKCC1 by its phosphorylation via the SPS1-related proline/alanine-rich serine-threonine kinase (SPAK). Postulated to be an inflammation-dependent dysregulation of ion flux and water movement, rates of CSF secretion increased acutely, remained elevated for up to 7 days, and contributed to the development of early ventriculomegaly in PHH [32]. Inhibition of TLR4, NF-κB, SPAK, or NKCC1 attenuated the high CSF production in this rodent model of PHH [32]. The role of TLR4 in this process raises the possibility of a contribution to other etiologies such as PIH where many PIH-associated bacteria have been shown to be TLR4 agonists [5].

The workshop revealed significant overlap between mechanisms identified in different models of PHH. For example, TRPV4 activation and the TLR4-NF-κB inflammatory pathway regulates not only NKCC1 activity, but also intracellular calcium levels. In the lungs, TRPV4-mediated calcium influx strengthens the innate defense responses of epithelial cells to LPS (lipopolysaccharides from gram negative bacteria) [92], although the early alveolar macrophage responses to LPS need to be counter-balanced by a subsequent reduction in TRPV4 activity to prevent an excessive inflammatory response [93]. Within a porcine CPe monolayer cell culture, the pro-inflammatory transcription factor, NF-κB, rapidly increased the CPe membrane conductance with similar kinetics to TRPV4 agonists, while other cytokines (IL-1β, TNF-α) can downregulate TRPV4 after 24 h. This time course suggests that NF-κB might drive an early influx of calcium within the CPe, which could be downregulated at later timepoints by CSF cytokines. If so, appropriately timed pharmacologic inhibition of TRPV4 might be a therapeutic approach to limit the later phase of inflammation [11]. Moreover, TLR4-dependent activation of NKCC1 can also regulate activation of TRPV4 in cerebral edema in a model of traumatic brain injury, which suggests that there may be additional overlapping mechanisms of regulation between NKCC1 and TLR4 [94]. These studies shed light on the complex nature of the inflammation within the CPe and demonstrate the necessity for further collaboration to develop new therapeutics that target specific harmful components of the innate immune response or limit particular phases of the inflammatory response.

### Where is the field heading, what do we need to know?

Recent research discoveries have increased our understanding of the cellular mechanisms by which IVH disrupts CPe function and leads to PHH. However, several questions remain to be addressed before novel treatments for hydrocephalus can be developed.

First, a full characterization of the changes in fluid and electrolyte transport in the CPe and perhaps other tissues during the course of hydrocephalus development across etiologies is lacking but will be necessary for a strong foundation to delineate targetable upstream mechanisms. For example, overexpression of NKCC1 via AAV in the choroid plexus attenuates ventriculomegaly in a model of obstructive hydrocephalus [50]. This could be in part due to the modulation of ion flux in the presence of increased co-transporters which would provide a novel avenue for therapeutics. Conversely, if functional expression of NKCC1 via phosphorylation increases the rate of ion flux across the choroid plexus, further elucidation of this process could lead to methods to decrease CSF hypersecretion [32].

Second, we need to determine which components of blood are most pathogenic and inflammatory. Although blood can cause a physical impediment that leads to obstructive hydrocephalus, there is a consensus that inflammation plays a role in the pathogenesis of PHH. After IVH, hemosiderin persists in the ventricular lining, facilitating toxic iron pathophysiology, promoting ferroptosis and inflammation, and thus damaging developing neural cells including oligodendrocytes, beneficial microglia and ependymal cells [6]. In adults with IVH, blood and cell-free hemoglobin activate cytotoxic, oxidative and inflammatory pathways leading to tissue damage and limiting spontaneous recovery of CSF dynamics. Multiple candidate molecules have been postulated, most notably related to breakdown products of red blood cells (e.g., heme and iron) as well as plasma clotting factors (e.g., thrombin). Metabolites of hemoglobin have pro-inflammatory effects on microglia, and macrophages, activating a significant immune response. Methemoglobin has been shown to be a specific ligand for TLR4 [95], which might represent a common etiology for inflammation in other types of hydrocephalus such as post-infectious hydrocephalus. Additionally, hepcidin is a major regulator of iron activity and transport in the brain. It is expressed mainly in astrocytes but can also direct iron accumulation in neurons, and is known to be present in endothelium, pericytes, microglia and throughout the ventricular system.

Third, during this workshop, and in the field, there is an emphasis on inhibiting inflammatory processes associated with PHH. As a result, there is less focus placed on which aspects of inflammation are reparative and necessary. It is likely that in time, it will be possible to delineate between adaptive and maladaptive responses and target them accordingly. For example, neural-immune cross talk is an essential component of health and physiology but can become maladaptive, especially in a brain primed by pre-existing inflammation or prior insult [96]. Immune plasticity altered by hydrocephalus may also have long-term effects. The persistent presence of soluble inflammatory mediators (i.e., chemokines and cytokines) are capable of not only disrupting the blood-brain barrier, ventricular system and choroid plexus directly, but also facilitate the migration and trafficking of macrophages, monocytes, neutrophils, and T-cells. Additional cytokines and chemokines produced by these circulating immune cells can move across barriers, impair function, and further increase immune cell movement in the brain [5]. These events compound the activation and stimulation of resident glia and propagate the toxic inflammatory  microenvironment defined by hydrocephalus. Cumulatively, these events can lead to profound structural and functional changes, aberrant connectivity and disrupted neural networks leading to lasting neurological sequelae including deficits in cognition that require further study in all forms of hydrocephalus.

Fourth, fundamental aspects of CSF regulation during normal development are still debated. It is universally accepted that the CPe produces a significant amount of CSF, but it is unclear what proportion of the total CSF it produces and whether this fluctuates within individuals. Following cauterization of the ChP, extra-choroidal CSF production is presumed to arise from the leptomeningeal and parenchymal vasculature [97].

Finally, clarifying the sites of CSF reabsorption remains controversial. The arachnoid granulations have long been postulated as the primary site of CSF reabsorption in humans. However, the apparent lack of arachnoid granulations in infants and rodents question this dogma. The likely dominant sites of CSF drainage in rodents, pertinent to many models of PHH, include meningeal lymphatic vessels (dorsal and ventral sites) as well as perineural and perivascular efflux to lymphatic sites (with arguable proportions of CSF drainage depending on e.g., head position). Notably, the glymphatic system has emerged as an important contributor to fluid movement inside the brain . This highly polarized CSF transport system facilitates the clearance of neurotoxic molecules through a brain-wide network of perivascular pathways [98–104]. This glymphatic clearance is dependent on AQP4 located on astrocyte vascular endfeet for exchange of solutes [98, 105]. There have also been reports of CSF clearance via the choroid plexus [50,106].

## Ependyma

Similar to the emerging role of the choroid plexus in hydrocephalus, the involvement of ventricular ependyma is an active area of research across multiple etiologies of hydrocephalus and may provide new targets for therapeutic interventions. During this session presentations were focused on ependyma development, plasticity, and injury in the context of hydrocephalus [107]. The ependyma is a single-layer of ciliated polar epithelial cells that line the cerebral ventricles, aqueduct, and central canal of the spinal cord [62]. Ependymal cells create a barrier between the CSF in the ventricles and the brain parenchyma and have important functions related to neurodevelopment, morphogenesis, and neurophysiology [108]. New studies consistently highlight the importance of ependymal cells in the development of hydrocephalus [109–111]. Indeed, at the workshop researchers reported dysregulation of the ependymal lining leading to hydrocephalus, concomitant with mechanical strain via increased intracranial pressure and ependymal denudation leading to reactive astrocyte replacement in the ventricular lining often called a glial scar. Moreover, in PHH and other etiologies of hydrocephalus ependymal cell alterations are precipitated by inflammation. Interestingly, studies that therapeutically reversed inflammatory processes affecting the ependyma, in part diminished the development of hydrocephalus. In total, this session highlighted ependymal cells as abnormal across different hydrocephalus etiologies and underscored the importance of elucidating mechanisms underlying ependymal involvement in the development and progression of hydrocephalus to assist in future efforts to develop non-surgical therapies.

### Ependymal development and plasticity

During this section of the workshop, exciting studies concerning ependymal development and plasticity were a prevalent topic of discussion. Differentiation of ependymal cells from radial glial cells of the neuroepithelium follows a precisely controlled temporal-spatial pattern throughout the ventricular system [112, 113]. In humans, the ependymal cells begin to line the neural tube by week 25 of gestation [114]. Studies in animals suggest that radial glial cells specified during embryogenesis give rise to adult neural stem cells and ependymal cells through symmetric and asymmetric divisions across the embryonic neuroepithelium [115–117]. Geminin family genes play a role in early specification of ependymal cells, in addition to the transcription factor Nuclear factor IX (NFIX) [118, 119] by activating transcription factor Foxj1. Foxj1, the DNA-binding forkhead transcription factor, is a master regulator of the ciliogenesis program. Maintained expression of FoxJ1 initiates the generation and docking of multiple basal bodies and motile cilia formation [111,120−122]. Formation and polar organization of motile cilia patches on the apical surface of ependymal cells marks completion of ependymal cell maturation [116, 123].

Along the walls of the ventricles, ependymal cells organize into rosette, pinwheel-like patterns with neural stem cells at their center [124, 125]. Ependymal cells are held together by adherens junctions, which maintain the cytoarchitecture integrity of the ventricular wall and stem cell niche [126–128]. In this arrangement, ependymal cells influence self‐renewal and differentiation decisions of the neural stem cells they surround [129–132]. Ependymal cells generally do not divide in the mature intact brain. However, in times of injury (e.g. stroke) ependymal cells can reenter the cell cycle and give rise to neuroblasts and astrocytes [133, 134]. This is interesting in the context of hydrocephalus given that there are consistent reports that mechanical strain via increased intracranial pressure and flattening of the ependyma lead to astrocytic cell replacement in the ventricular lining. As discussed above in the CPe section, NF-κB is upregulated in PHH in the ependyma.

### Ependymal alterations and observations in hydrocephalus

Researchers discussed PHH as a severe manifestation of encephalopathy of prematurity as the inflammatory biology of ependymal cells specific to infants under six months of age represents an important susceptibility factor. However, the vulnerability of mature ependymal cells could also contribute to instability and impact ventricular dynamics. In PHH, injury to the ventricular wall is prominent such that high intracranial pressure and mechanical strain at the ventricular surface results in flattening or loss of ependymal cells and compression of the surrounding cerebral cortex and white matter [6, 44, 108]. Interestingly, ependymal cells of the ventricular walls can rapidly de-differentiate in response to injury involving IKK2 (inhibitor of nuclear factor kappa-B kinase 2) inhibition resulting in hydrocephalus [111]. As such, ependymal cell transformation leading to ventricular breakdown, ependymal cell basolateral membrane specialization, and ependymal regeneration are all important considerations in the study of PHH. Notably, yes-associated protein (YAP), the downstream effector of the Hippo pathway, is critical for regulating the normal generation of ependymal cells along the ventricular surface. Disruption to YAP signaling precipitates a loss of junctional integrity during ependymal maturation, resulting in the development of hydrocephalus and abnormal neurogenesis [135].

Conversely, concepts that involve using radial glial stem cells to generate ependymal cells with NFIX were also discussed. NFIX is central to the development of the ependymal layer in the lateral ventricles and expression of ependymal cell-specific markers is delayed in the absence of NFIX [119]. NFIX-deficient mice have macrocephaly with ventriculomegaly, abnormal ependymal cell morphology, and cell loss. Importantly, FoxJ1 is a target for NFIX-mediated transcriptional activation [119], linking key concepts between motile cilia and ependymal sessions of this workshop.

### Ependymal therapeutic strategies & windows of opportunity

The degree to which the ependymal layer can respond, and potentially recover following surgical and non-surgical intervention was also discussed in detail at the workshop. Medical therapies targeting inflammation and reducing cellular and molecular strain on the ependyma were introduced in the context of PIH and PHH of prematurity. Therapies targeting YAP, FoxJ1, neural-immune interactions and immune modulation were emphasized. To begin, therapeutic strategies that target or support YAP were discussed at great length, as YAP dysregulation is a common element in many models and studies of hydrocephalus [135–137]. Like inflammation, YAP signaling perturbations may be a common underlying mechanism that links congenital and acquired hydrocephalus, including PHH. YAP/TAZ maintain ependymal cells in the ventricular lining. In a mouse model of fetal hemorrhagic hydrocephalus, decreased YAP expression depletes ependymal cells and leads to aqueductal stenosis [135]. Indeed, YAP overexpression rescues the ependymal cells and attenuates the development of hydrocephalus [135]. In the context of PHH and inflammation, there is an intimate connection between YAP and lysophosphatidic acid (LPA) [138]. It is generally thought that LPA is a lipid signaling molecule circulating in serum within platelets or bound by carrier molecules such as albumin [138, 139]. While LPA has been shown to have roles throughout multiple organ systems [140], it has recently been demonstrated to be a pro-inflammatory molecule implicated in CNS disorders including multiple sclerosis [141], traumatic brain injury [142, 143], and hydrocephalus [135, 139, 144]. Interestingly, LPA has a critical role in upstream regulation of YAP. LPA reduces YAP expression, and analyses of LPA receptor null mice have identified LPA1 and LPA3 as key mediators of PHH [139]. Similarly, pharmacological blockade of LPA1 prevents PHH in LPA injected animals, supporting a role for LPA receptor antagonism in the prevention of PHH.

Consistent with the theme of YAP stability and attenuation of inflammation, diminished ependymal YAP expression with concomitant increase in the expression of glial fibrillary acidic protein (GFAP), a marker for astrocytes, has been shown in a rat model of PHH of prematurity [53]. Notably, delayed systemic administration of erythropoietin (EPO) plus melatonin (MLT) therapy reverse these cellular and molecular changes concomitant with attenuation of macrocephaly, reversal of ventriculomegaly, reduction in intracranial pressure and mitigation of white matter injury in PHH of prematurity. The actions of EPO + MLT are multifold. In addition to attenuating abnormalities in YAP, they also reduce global markers of neuroinflammation and impact the function of the motile cilia on ependymal cells. Like EPO + MLT, therapies targeting the metalloprotease ADAM10 in PHH and PIH and their relationship to neuroinflammatory state, immune activation, junctional proteins, and ependymal cell health was also discussed. Notably, activation of ADAM10 and hence its cleavage of the cell-junction protein, N-cadherin impairs differentiation of cells in the subventricular zone after PHH and catalyzes periventricular white matter injury [145]. Thus, pharmacologic inhibition of ADAM10-mediated N-cadherin cleavage may be a novel beneficial therapeutic approach in PHH.

## Cilia

Building a comprehensive view of the diverse cellular injury related to hydrocephalus enables basic and translational researchers to identify new targets for therapeutic development as well as to evaluate rescue by new therapeutic regimens. During the workshop, presentations by basic scientists covered factors that influence cilia formation and the role of especially motile cilia in central nervous system development [146–149]. These presentations were followed by a discussion of recent preclinical data from animal models demonstrating cilia injury in the context of congenital hydrocephalus, intraventricular hemorrhage, and PHH of prematurity. This session highlighted ependymal motile cilia as abnormal across different hydrocephalus etiologies and encouraged future studies to examine ependymal motile cilia injury and functional rescue following novel pharmacological interventions.

### Diversity of ventricular cilia

Cilia are organelles that extend outward from the apical surface of cells and are involved in sensory perception and the transport of fluid within the ventricular system [146, 149]. Cilia classification is based on axoneme structure. The ciliary axoneme is the long, delicate, membrane bound projection arising from the basal body, which anchors the cilium to the apical surface of the cell. The absence or presence of a central pair of singlet microtubules, encompassed by nine radially arranged microtubule doublets, informs the classification of cilia as 9 + 0 (non-motile) or 9 + 2 (motile) cilia, respectively [150, 151]. 9 + 0 cilia or non-motile cilia function as chemosensors, mechanosensors, and positional sensors to maintain homeostasis [49, 96], and can be found on the apical surface of radial glial (immature ependymal) cells lining the neonatal ventricle [116]. CPe epithelial cells develop 9 + 0 cilia patches that exhibit transient motility during the neonatal period [152, 153]. In adulthood, neural stem cells in the subventricular zone extend a solitary 9 + 0 non-motile cilium into the CSF [124, 154]. During the workshop, discussion centered on the 9 + 2 ependymal motile cilia that grow in tufts on the apical surface of mature ependymal cells that line the ventricles, as mounting evidence supports their involvement in PHH of prematurity pathophysiology.

### Ependymal motile cilia function & development

Motile 9 + 2 cilia ultrastructure is highly conserved across eukaryotes, however, notable differences in cilia length and distance between cilia on multi-ciliated cells are observed [155]. Intriguingly, the character of cilia movement and coordination of movement patterns across neighboring cilia varies significantly between organisms [155]. In smaller vertebrates such as zebrafish, single motile cilia found on ventricular ependymal cells exhibit rotational movement that functions to propel CSF in organized patterns, critical for ventricular system development in this species [156]. In contrast, mature ependymal cells lining the mammalian ventricular system possess tuffs of 9 + 2 motile cilia that beat in concerted whip-like motions to produce organized streams of CSF movement [154, 157−160]. The planar beating of these 9 + 2 cilia is generated by the sliding action of inner dynein arms, outer dynein arms, and the nexin-dynein regulatory complexes that bridge neighboring radial microtubules [161, 162]. The near-wall fluid dynamics created by tuffs of motile cilia across neighborhoods of ependymal cells play an important role in neural development, and support brain homeostasis by directing neuroblast migration, clearing debris and toxins, promoting the distribution of factors produced by the choroid plexus, and maintaining neural stem cell proliferation and the neurogenic niche [163–166].

The occipital to frontal progression of 9 + 2 multi-ciliogenesis along the ventricular wall occurs late in fetal development, taking place in humans from 34 gestational weeks to 10 days after birth at term [107]. For comparison, multi-ciliogenesis of ependymal cells in rodents occurs from postnatal day 1 to 15 [107, 116, 167]. 9 + 2 cilia motility requires the proper biogenesis and assembly of a complex array of cilia components. Ependymal motile cilia formation is predominantly dependent on the expression and maintenance of the DNA-binding forkhead transcription factor, Foxj1 [111, 121, 122, 168]. During ciliogenesis Foxj1 acts in coordination with the Regulatory factor X (Rfx) family of stabilizing transcription factors as a master regulator, activating a constellation of genes responsible for initiating intraflagellar transport and cilia assembly in terminally differentiated ependymal cells [169, 170]. Foxj1 activation precipitates basal body docking to the apical surface of the ependymal cell, essential for the generation of multiple motile cilia [121, 122]. Upstream of FoxJ1 activation, Geminin family members GemC1 and Mcidas form complexes with E2F4/5 transcription factors, leading to centriole amplification and subsequent production of multiple cilia [110, 118, 171−174]. Newly docked basal bodies first display unorganized orientations, and then reorient in a common direction through the coupling of hydrodynamic forces and the expression of Vangl2, the planar cell polarity protein [123, 175−177]. Effective cilia-generated fluid flow depends on this planar polarization of basal bodies from which motile cilia grow [176, 178].

### Connecting motile ciliopathy to hydrocephalus

Investigations utilizing mutant mouse models to alter the expression of ciliogenesis regulatory factors are associated with the phenotypes related to hydrocephalus. Inhibition of Foxj1-orchestrated ependymal motile ciliogenesis appears to precipitate hydrocephalus. In a conditional knockout mouse model, tamoxifen-induced knockout of Foxj1 expression in ependymal cells at postnatal day 14 resulted in loss of motile cilia and ventriculomegaly after two weeks [111]. Further, in mouse models employing targeted deletion of Foxj1 in mouse embryonic stem cells, 9 + 2 ependymal motile cilia formation is prevented resulting in macrocephaly and progressive ventriculomegaly in neonatal mice [120, 179]. Moreover, in a clinical study, six individuals with hydrocephalus were identified through whole-exome or whole-genome sequencing to be heterozygous for *de novo* mutations in Foxj1 [180].

A multitude of mutant mouse models with altered expression of proteins essential for cilia assembly, structure, or apical organization exist, including: Mdnah5 [181], Ift88 [182], Hy3 [183, 184], Celsr2 and Celsr3 [176], Pkd1 and Pkd2 [185, 186]. Each of these models demonstrates impaired ciliary motility and subsequent development of a hydrocephalus phenotype. For example, the *Ccdc39* gene that encodes protein important for functional arrangement of the inner dynein arm and nexin-dynein regulatory complex within the axoneme is essential for ciliary motility [187]. Mutation of the Ccdc39 gene in mice results in shorter ependymal motile cilia, impaired ciliary beating, altered CSF flow, and development of neonatal ventriculomegaly [109].

Preclinical models of PHH of prematurity have identified injury to ependymal motile cilia. In a rat model of PHH of prematurity, intraventricular injection of lysed red blood cells, hemoglobin, or iron on postnatal day 7, during cilia maturation, all resulted in ventriculomegaly and loss ependymal motile cilia at 24 h following injection [188, 189]. In another rat model that recapitulates the inflammatory neural microenvironment of preterm infants [190, 191], IVH was induced at the beginning of ciliogenesis on postnatal day 1 through bilateral intraventricular injection of lysed red blood cells. This approach resulted in ventriculomegaly, progressive macrocephaly and microstructural neural injury, concomitant with morphological changes to ependymal motile cilia, as revealed by scanning electron microscopy on postnatal day 21 [53]. Collectively, these studies demonstrate that impaired ependymal cilia motility significantly derails ventricular homeostasis leading to the development of hydrocephalus in animal models.

### Future directions and therapeutic avenues

Recent investigations have given us a glimpse into the complex process of cilia development in the ventricular system, in addition to revealing the important roles these structures play in brain homeostasis. Although researchers have begun to examine injury to cilia in the context of IVH and in the development of PHH of prematurity, many questions remain unanswered. For instance, while loss of ependymal motile cilia has been described following exposure to blood products, the specific molecular mechanisms involved resulting in this effect are unknown. Inflammation affects the expression of Foxj1 [111], however the mechanisms still need to be clarified in preclinical PHH models. During the workshop, gaps in knowledge were identified including whether there exists a peak stage in vulnerability to develop PHH of prematurity injury along the time course of ependymal motile cilia maturation, and if ependymal motile cilia across spatially distinct ventricular regions are equally vulnerable to injury. It is important to also mention that notable differences are emerging between mouse models and human hydrocephalus including (1) motile ciliopathies in humans are rarely accompanied by hydrocephalus, and (2) genes associated with hydrocephalus may also involve altered neural development that likely contributes to hydrocephalus by other as yet unidentified mechanisms [192–194]. Addressing all of these points will directly inform future efforts to develop therapeutic strategies suitable for PHH of prematurity. Following the fruitful discussions that took place during the workshop, it was clear that injury to ependymal motile cilia is likely one of the many facets of PHH pathophysiology.

## Emerging areas of study

Two talks focused on emerging areas of study that may intersect with the development of select hydrocephalus etiologies. The first discussed the development of the human germinal matrix and its implications in neurodevelopmental disorders. During brain development, perturbations of the interplay between neural progenitor cell proliferation, young neuron migration, and neurovascular development can cause disease. In a number of recent studies, the interplay between neural progenitor cells and blood vessel cells has been evaluated [195, 196] and disrupting the contacts has altered progenitor cell fate [195]. It is anticipated that similar, complex relationships exist between the neural progenitor cells and vasculature of the germinal matrix. The germinal matrix is located along the wall of the lateral ventricles within the ganglionic eminence. It is present transiently during gestation with its thickness peaking at 24 weeks gestation. By 36–37 weeks it is nearly absent [21, 27−30]. While present, the germinal matrix is a highly vascularized area with a high density of neural and glial progenitor cells [197, 198] as well as young neurons. These young neurons are highly proliferative and migratory, generating cortical GABAergic interneurons and oligodendroglia [199, 200]. In the extreme example of hemorrhage of the germinal matrix, these contacts are likely disrupted and may help explain some of the neurodevelopmental conditions associated with PHH of prematurity.

The second talk focused on the role of microglia in remodeling brain circuits. Microglia are present within neural circuits and play a role in synaptic pruning during development as demonstrated in the developing retinogeniculate system [201]. Blocking the complement-mediated synaptic engulfment by microglia results in defects in synaptic pruning [201]. Much of the neurodegenerative disease field has been focused on neuronal cell death and axonal degeneration [202]. However, recent evidence suggests aberrant pruning occurs at early time points [202], prior to neuronal cell death and axonal degeneration, in a model of autoimmune encephalomyelitis. At this time, reactive microglia were visualized engulfing synaptic material, mediated by complement receptor 3, and inhibition of synaptic complement 3 prevented synaptic pruning and preserved visual acuity in the model. For hydrocephalus, this work may have implications for visual, cognitive, and motor dysfunction in the presence of myelin sheath injury [203, 204] and reactive gliosis which has been identified in both human and animal models of hydrocephalus [205].

## Opportunities and challenges in cell therapies

Many of the workshop discussions were focused on potential drug or molecular targets and treatments for hydrocephalus. However, the rapid increase in cell therapy trials for other conditions is paving the way for their potential use in hydrocephalus. The final session was therefore a discussion of the opportunities and challenges of using cell therapies to prevent, treat, or improve outcomes in hydrocephalus. After more than half a century of bone marrow transplantation, followed by Nobel prizes for Gurdon and Yamanaka less than 10 years ago, cell therapies have become a new frontier for patients with hydrocephalus and other disabling conditions. Given their position between molecules and organs in the hierarchy of biological organization, cells represent a conceptual “sweet spot” in the clinical toolkit with the potential for greater clinical trial success than molecules or drugs, and better supply than organs. Moreover, the pipeline for cell therapies is growing rapidly. At the time of the Hydrocephalus Association workshop in November 2019, ClinicalTrials.gov listed over 2,500 stem cell interventional trials, not including those involving bone marrow or hematopoietic stem cells, and the California Institute for Regenerative Medicine was actively sponsoring 56 clinical trials addressing 35 different unmet medical needs.

Unfortunately, the promise of cell therapies comes with risk from the rise of “mom and pop” clinics and online marketing for unapproved cell therapies, both nationally [206] and globally [207]. While analogies can be drawn to the period after the first bone marrow transplants, the magnitude of marketing for these generally unproven, often questionable, and potentially harmful cell therapies is startling. Regardless of one’s perspective on this situation, it is undeniable that desperate patients and families will consider unproven cell therapies if there is a chance of alleviating a loved one’s pain and suffering.

For now, proven cell therapies for hydrocephalus are non-existent. Hydrocephalus poses many challenges for cell therapies, including (but not limited to) disease complexity, lack of straightforward cell replacement strategies, defective/damaged cells being non-causal, involvement of cell niches with limited regenerative capacity, and temporal factors (e.g. prenatal onset). Based on prior studies in young babies with congenital hydrocephalus [208], a compassionate use study led by Dr. Joanne Kurtzberg at Duke University involving sibling or autologous umbilical cord blood infusions is available to children with one of seven brain disorders, including hydrocephalus. Other cell-based trials for hydrocephalus were not found in the ClinicalTrials.gov database.

While cell therapies for hydrocephalus lag considerably behind surgical interventions, devices, and drugs, dramatic advances using stem cells offer hope and opportunity for meaningful hydrocephalus studies and applications. Induced pluripotent stem cells (iPSCs) [209] enable basic studies and potential applications tailored to individual patients. When coupled with the derivation of specific human cell types and organoids relevant to hydrocephalus, such as choroid plexus epithelial cells [210], choroid plexus organoids [211], and brain organoids [212], novel and rational approaches for hydrocephalus modeling, drug screens, and cell therapies using human cells become feasible. These precedents provide the foundation for deriving other human cell types and tissues relevant to hydrocephalus that remain to be described, such as ependyma and arachnoid, arachnoid villi and granulations, and cerebral lymphatics. Together with gene delivery technologies, drug delivery systems that take advantage of CSF access to the hydrocephalic brain as well as cellular reprogramming strategies to replace damaged cells, such as ependymal cells [110], can now be envisaged.

Nonetheless, while reasons abound for long-term optimism, proven cell therapies for hydrocephalus do not exist. The near-term is therefore fraught with risks for hydrocephalus patients and their families, who are strongly advised to navigate these risks in consultation with reputable and trusted medical advisors when considering any cell therapy for hydrocephalus.

## Conclusion

Hydrocephalus is a devastating condition that affects people of all ages. With no effective non-surgical therapies, improved understanding of common pathophysiological mechanisms may yield therapeutic and technical innovations (Fig. 1). The Hydrocephalus Association *Driving Common Pathways: Extending Insights from Posthemorrhagic Hydrocephalus (PHH)* workshop identified many important areas for future research and collaborative team-science to accelerate the development of novel therapies and key mechanistic insights aimed at reducing the global burden of hydrocephalus and associated sequelae.

## Data Availability

Not applicable.
